# Predictive Nephrotoxicity Profiling of a Novel Antifungal Small Molecule in Comparison to Amphotericin B and Voriconazole

**DOI:** 10.3389/fphar.2020.00511

**Published:** 2020-04-24

**Authors:** Nadeeka S. Udawatte, Sung Wook Kang, Yue Wang, Thiruma V. Arumugam, Chaminda J. Seneviratne

**Affiliations:** ^1^ National Dental Centre Singapore, Oral Health ACP, Duke-NUS Medical School, Singapore, Singapore; ^2^ Department of Physiology, Yong Loo Lin School of Medicine, National University of Singapore, Singapore, Singapore; ^3^ Institute of Molecular and Cell Biology, Agency for Science, Technology and Research (A*STAR), Singapore, Singapore

**Keywords:** antifungal induced nephrotoxicity, Amphotericin B, Kim-1 (Kidney injury molecule-1), Clusterin, small molecule

## Abstract

**Background and Purpose:**

*Candida albicans* is the major fungal species associated with superficial mucosal infections such as oral candidiasis as well as systemic mycoses with high morbidity and mortality. On top of the rising drug resistance, currently available antifungal agents have significant adverse effects. Nephrotoxicity is the major treatment complication associated with antifungal agents.

Recently, we discovered a novel antifungal small molecule SM21 with promising antifungal activity. The present study aimed to comparatively evaluate the *in vivo* and *in vitro* nephrotoxicity of SM21 comparing with Amphotericin B and voriconazole.

**Experimental Approach:**

Nephrotoxicity of SM21 and its analogue were comparatively evaluated with Amphotericin B (AmB) and voriconazole. Immortalized human kidney proximal tubule epithelial cells (HK-2) were used for *in vitro* analysis of nephrotoxicity using cytotoxicity assays and qPCR gene expression analysis (Kim-1/*HAVcr-1, CASP3*). Sprague Dawley (SD) rat model was used to evaluate the nephrotoxicity *in vivo* using classical (SCr and BUN) and next-generation kidney injury urinary biomarkers (Kim-1, CLU, ALB, NGAL, β2M, and Cys C) alongside histopathological and immunohistochemical standards.

**Key Results:**

AmB treatment showed a stronger cytotoxic impact on HK-2 viability and gene expression of cell death markers (Kim-1/*HAVcr-1, CASP3*) compared with SM21 and SM21 analogue *in vitro* (P < 0.01). In vivo data further demonstrated that SM21 did not significantly increase classical as well as novel nephrotoxic biomarkers, and minimal renal tubular necrosis and abnormalities were observed (15 mg kg^−1^ BW/day).

**Conclusions and Implications:**

SM21 had a significantly better safety profile in terms of nephrotoxicity with no major tubular epithelial abnormalities observed in kidney cells and no augmentation of kidney injury biomarkers compared to AmB. Kim-1 and CLU were the most sensitive biomarkers for detection of AmB-induced kidney damage. Future clinical trials should consider inclusion of these novel biomarkers as early indicators of acute kidney injury in antifungal-induced nephrotoxicity.

## Introduction

Invasive fungal infections are increasingly common in the nosocomial setting (Perlroth et al., 2007). *Candida* species are the most common fungi causing invasive disease in humans and the fourth-most prevalent pathogen of nosocomial bloodstream infections (Wisplinghoff et al., 2004). Of the *Candida* species, *Candida albicans* is the most prevalent pathogen, causing approximately 400,000 life-threatening systemic infections worldwide each year in severely immunocompromised patients (Dantas Ada et al., 2015). Treatment options for *Candida* infections are limited due to drug-related toxicity (Ostrosky-Zeichner et al., 2010; Pfaller, 2012) and the emergence of antifungal resistant strains (Whaley et al., 2017). Therefore, the development of new antifungal agents is a medical priority.

Evaluation of drug-related toxicities is an important milestone in the development of new antifungal agents. Approximately, 92% of the new compounds fail in the drug development process due to toxic side effects (Hill and Rang, 2012). Drug-induced kidney injury is one among the reasons for the compound attrition in drug development (Kola and Landis, 2004; Fuchs and Hewitt, 2011). It is a common adverse effect of Amphotericin B (AmB), which is regarded as the “gold standard” antifungal agent (Kuznar and Baglin, 2015). Therefore, the nephrotoxic effect of existing antifungal agents, particularly of AmB, has been extensively studied using *in vitro* and *in vivo* models (Van Etten et al., 1993). Combination therapy with caspofungin and voriconazole or liposomal amphotericin B (L-AmB) are considered as another first-line choice of antifungals in critically ill patients with invasive candidiasis (Keane et al., 2018). Although the newer antifungals L-AmB and caspofungin have considerably lower nephrotoxicity; infusion-related adverse reactions and renal dysfunction are still common (Olson et al., 2008; Felton et al., 2014). Therefore, it is imperative to comprehensively investigate the nephrotoxic effect of any new antifungal agent before clinical trials.

We recently discovered a novel antifungal small molecule SM21, following a high-throughput screening of a library with 50,240 small molecules. (Wong et al., 2014). SM21 demonstrated excellent antifungal activity against *Candida* species, including isolates resistant to existing antifungals such as azoles, caspofungin, and AmB. SM21 has a broad spectrum of activity against *Candida* species including azole-, caspofungin-, and amphotericin B-resistant strains. Additionally, SM21 did not exhibit antibacterial activity even at 10 times the effective concentration for fungi, akin to other antifungal agents and the Minimum Inhibitory concentration (MIC) of SM21 is comparable to that commonly used antifungals such as AmB (Wong et al., 2014). Subsequently, we identified the mechanism of action of SM21, which targets fungal-specific mitochondrial proteins (Truong et al., 2018). Moreover, no detrimental effects were observed with SM21 in a candidiasis mice model (Wong et al., 2014). As the next step of development, it is important to comprehensively examine the safety aspect of SM21 pertaining to nephrotoxicity, as mentioned earlier.

Previous studies have used the classical nephrotoxic biomarkers such as serum creatinine (SCr), blood urea nitrogen (BUN) alongside histopathology standards to evaluate nephrotoxicity. However, due to discrepancies in the estimation of nephrotoxicity between the preclinical and clinical stages (Fuchs and Hewitt, 2011; Hill and Rang, 2012), the Nephrotoxicity Working Group of the Critical Path Institute Predictive Safety Testing Consortium (PSTC) proposed to explore 23 new renal biomarkers (Sistare et al., 2010) to improve sensitivity and specificity. These biomarkers were systematically evaluated in multiple mechanistically distinct animal models of kidney injury with well-established nephrotoxicants (Dieterle et al., 2010; Vaidya et al., 2010a). Seven renal safety biomarkers [kidney injury molecule (Kim-1), albumin (ALB), total protein, β2-microglobulin (β2M), cystatin C (Cys C), clusterin (CLU), and trefoil factor-3] were qualified for limited use in nonclinical and clinical drug development following submission of drug toxicity studies and analyses of biomarker performance for the FDA (U.S. Food and Drug Administration) and EMA (European Medicines Agency) by the PSTC Nephrotoxicity Working Group. However, there are no studies in the literature that has used these panel of novel biomarkers to evaluate antifungal induced nephrotoxicity.

In the present study, we have used the foregoing few novels and classical nephorotoxicity biomarkers alongside histopathological and immunohistochemical standards to evaluate the nephrotoxicity of SM21 in comparison with AmB and voriconazole, employing *in vitro* and standard *in vivo* models. The novel data will be important for the drug development process of SM21 as a novel antifungal agent. Moreover, the present study has laid the pioneering framework to evaluate comparative nephrotoxicity in the first-line choices of few antifungal agents (AmB and voriconazole) for invasive candidiasis using novel kidney injury biomarkers.

## Methods

### Antifungal Agents

AmB (Cat # A9528) and voriconazole (Cat # PZ0005) were obtained from Sigma-Aldrich (St. Louis, MO, USA). SM21 (PubChem CID: 5351098- [4-[2-(2,6-di*tert*-butylpyran-4-ylidene)ethylidene]cyclohexa-2,5-dien-1-ylidene]-dimethylazanium;perchlorate]) and SM21 analogue (PubChem CID: 2147408 shares >0.9 2D similarity Tanimoto coefficient score- [4-[2-(2,6-di*tert*-butylpyran-4-ylidene)ethylidene]cyclohexa-2,5-dien-1-ylidene]-dimethylazanium]) were purchased from ChemBridge (San Diego, CA, USA) and Mcule (Budapest, Hungary); respectively.

### *In Vitro* Nephrotoxicity Model to Comparatively Evaluate SM21 With Current Antifungal Agents

#### Cell Culture and Drugs Preparation

*In vitro* model of HK-2 cells (ATCC^®^ Cat# CRL2190™, RRID: CVCL_0302), an immortalized human proximal tubule epithelial cell line was used for the study as previously described (Ryan et al., 1994). Briefly, HK-2 cells were cultured in 75 cm^2^ cell culture flasks containing keratinocyte serum-free medium (Gibco, Cat# 17005-042) supplemented with bovine pituitary extract (0.05 mg ml^-1^) and human recombinant epidermal growth factor (5 ng ml^-1^) and penicillin (100 U·ml^−1^)/streptomycin (100 μg·ml^−1^; Gibco). Cells were cultured in a humidified atmosphere at 37°C with 5% CO_2_ and passaged twice per week. 100 mM stock solutions of SM21, SM21 analogue CID2147408, AmB, and voriconazole drug dissolved in 1% DMSO in PBS were serially diluted to obtain the required concentrations.

#### Evaluation of Cellular Cytotoxicity

HK-2 cells at a density of 2x 10^4^ cells per well in a 96-well plate platform were treated with five concentrations, namely, 0.1, 0.5, 1, 3, and 10 μM of each antifungal drug and incubated at 37°C in a 5% CO_2_ incubator for 24 h. Intracellular ATP concentration was determined by CellTiter-Glo^®^ Luminescent Cell Viability Assay (Promega, Cat# G7571) according to the manufacturer’s instruction (Promega, 2013). The cells were incubated for approximately 10 min under dark rotation (300 rpm) at room temperature until the cells were completely lysed and the readings were measured using a luminometer immediately. Each data point was evaluated in triplicates, independently five times using different cell passages; including blank and the vehicle control (cells treated with PBS in 1% DMSO). The resulting data were analyzed by calculating the arithmetic mean of the triplicates and then subtracting the blank value. The mean values of the treatments were then compared with the corresponding vehicle control to generate relative values that were used as the cell viability employing of logistic fitting of the dose-response CC_50_ curves calculated using GraphPad Prism 8 software.

#### Quantitative PCR Analysis of Nephrotoxic Biomarkers

HK-2 cells seeded in 6-well plates at a density of 2 × 10^5^ cells per well were exposed to concentrations of 1, 3, and 10 μM of each drug for 24 h. Cells were then harvested and total RNA was isolated using the RNeasy mini kit (Qiagen, Cat# 74104) with on-column DNase digestion according to the manufacturer’s instructions. The RNA concentration and quality were determined by NanoDrop Spectrophotometer. cDNA was generated from 1 μg RNA using the High Capacity cDNA Synthesis Kit (Life Technologies, Cat# 4368814). Quantitative PCR analysis of *HAVcr-1*(Sino Biologicals Cat# HP100847) and *CASP3* (Sino Biologicals Cat# HP100140) in each sample was performed in duplicates using Applied Biosystems real time PCR system (California, United States) in 20 μl reactions containing 2x master mix with SYBR Green (Bio-Rad, Cat# 4385618), cDNA, and of each primer. Amplification efficiencies of both the endogenous reference gene (H*prt-1*) and target genes were assessed using standard curves. Product specificity was examined by melting curve analysis and electrophoresis on a 5% polyacrylamide gel. Gene expression changes relative to untreated controls were determined by the 2^−ΔΔCt^ method (Livak and Schmittgen, 2001). Samples were amplified in duplicates and normalized against H*prt-1*(Sino Biologicals Cat# HP100005). Results were taken as mean fold change in mRNA expression of each treatment relative to control value as one.

### Evaluation of Comparative Nephrotoxicity *In Vivo*

To minimize the experimental variation that might be introduced by using animals from different strains, genders, or ages, male Crl : CD Sprague Dawley (SD) rats (4–5 weeks) weighing 200–300 g were purchased from Charles River Laboratories (Wilmington, MA, USA) (Strain Code # 400) for the toxicity studies. They were randomly divided into 5 groups as AmB, SM21, SM21 analogue, voriconazole, and vehicle control (saline), with each group comprising 9 (n=9) animals. The animals were housed two per cage in a controlled room (22°C, 12 h light/dark cycle) with food and water ad libitum for 1 week before the start of the experiment.

All animal-related procedures were conducted in an Association for Assessment and Accreditation of Laboratory Animal Care (AAALAC)—an accredited facility at the National University of Singapore, and the protocol (R17-0327) was approved by the Institutional Animal Care and Use Committee (IACUC). As per the IACUC guidelines, power analyses, i.e data from similar past experiments, were used to estimate the lowest number of animals required to reach statistically significant results.

Drug dosing solutions of 5 mg kg^-1^ body weight (BW) (low dose group) and 15 mg kg^-1^ BW (high dose group) were prepared daily and administered once per day by intravenous injection, for 5 consecutive days. Control groups received an equal volume of 0.9% physiological saline. AmB, which is known to cause *in vivo* nephrotoxicity, was used as a positive control and voriconazole, which has no nephrotoxic effect, served as a negative control. The rationale for the choice of doses was partially based on previous studies that found the dose of 15 mg kg^-1^ day^-1^ after 5 days of treatment produced some renal dysfunction, while avoiding overt kidney failure or death in animal models treated with AmB (Varlam et al., 2001; Journal and Nephrotoxicity, 2002). Likewise, when the principle of allometry is applied, the equivalent intravenous dose of AmB in humans corresponding to doses of 5 mg/kg and 15mg/kg in rats would approximate to doses that are routinely administered in current clinical practice guidelines (0.25–1.5 mg/kg). (Pharmacology of amphotericin B, 2019).

The animals were housed in individual metabolic cages during the study. Bodyweight, food, and water consumption were recorded daily. Urine samples were collected in 0–24 h interval on days 0 (before first administration of drugs), 1, 3, and 5 followed by centrifuged at 400 rpm for 5 min; immediately aliquoted and stored at −80°C until downstream analysis. The urinary volume was recorded on each time interval to measure the urinary creatinine (UCr) of the respective samples. At the end of day 5, the animals were sacrificed by CO_2_ asphyxiation and blood was collected by cardiac puncture once per animal. Blood samples were allowed to clot for 15 min, centrifuged for 10 min at 5,000 g to obtain the serum, and then aliquoted and stored at −80 °C until further analysis. Kidneys were removed and weighed. The kidney lobes were fixed in 10% neutral buffered formalin and embedded into paraffin. Kidney weights data were subjected to the linear regression and correlation was established with corresponding body weights using Prism’s correlation matrix.

#### Evaluation of Standard and Novel Nephrotoxic Biomarkers

The assay kits for the measurement of SCr (Abcam, Cat# ab65340), urinary creatinine (Invitrogen, Cat# EIACUN) and BUN (Invitrogen, Cat# EIABUN) were obtained. The experiments were performed according to the manufacturer’s instruction. The levels of SCr and BUN were normalized against the control groups. For urinary biomarkers assay, urine samples collected on days 0, 1, 3, and 5 were assessed using customized MILLIPLEX MAP Rat Kidney Toxicity Magnetic Bead Panel 1 and 2 (Merck, Cat# RKTX1MAG-37K and RKTX2MAG-37K; respectively). Kim-1 and CLU levels were analyzed using the rat kidney toxicity panel 1 assay kit. The levels of Cys C, NGAL (neutrophil gelatinase-associated lipocalin), ALB, and β2M were assayed using rat kidney toxicity panel 2. Briefly, the samples were diluted with the sample dilution buffer and then incubated with capture beads for 16–18 h at 4°C in the dark. The primary antibody for each biomarker and followed by streptavidin–phycoerythrin, were added and incubated at each separate step. The readings were measured by MAGPIX^®^ with xPONENT^®^ software. The Median Fluorescent Intensity (MFI) data were analyzed using a spline curve-fitting method for calculating analyte concentrations in samples. Duplicate samples with a CV value greater than 15% were rejected and any standard curve values with recovery outside the range of 80–120% were omitted from the curve fit process. Internal QC material was generated using standard curve material (two points) chosen to be on the linear part of the curve, and within- and between-plate precision was calculated. Test plates were subsequently accepted if QC material values fell within +2 SD of their calculated means. The intra- and inter-assay precisions were also calculated. The QC range given was based on a minimum of six assays run by at least three different operators in Merck, St Louis. The high/low values were obtained from +/- 35% of the mean. The quantity of biomarkers in the urine was presented after normalization, the individual biomarker concentration in ng/ml was simply divided by the respective UCr concentration (mg/ml) with the final normalized result measured in ng/mg (Adedeji et al., 2019).

#### Histopathological Examination of the Kidney

Kidney specimens were cut into 5 μm thick sections, stained with hematoxylin and eosin (H&E) (Sigma, Cat# MHS32 and E4009; respectively), and examined under light microscopy for semiquantitative analysis of the kidney sections. The kidneys were examined for tubular epithelial alterations (dilatation, desquamation, vacuolization, necrosis, and atrophy), interstitial inflammation, edema, and glomerular alterations. All histopathological parameters were graded according to the Critical Path Institute’s Predictive Safety Testing Consortium (PSTC) Nephrotoxicity Working Group (NWG) histopathology lexicon and assigned severity scores on a scale of 0–5 to grade pathological lesions, with 0 being no observable pathology, 1 minimal, 2 slight, 3 moderate, 4 marked, and 5 severe (International Society of Nephrology, 2012). The composite score for an individual animal was calculated as the highest ordinal score for any histopathologic process at any kidney site.

#### Immunohistochemistry of Kidney Sections

Kidney sections (5 μm) were prepared on formalin-fixed, paraffin-embedded tissue blocks, and mounted on glass slides. The sections were deparaffinized, rehydrated, and washed in PBS. Heat-induced antigen retrieval was achieved by autoclaving for 4 min in 10 mM citrate buffer, pH 6.0. For the detection of Kim-1 and CLU, kidney sections were treated with 0.1% trypsin for 2 min at 37°C, followed by washing in PBS and blocking with 5% goat serum for 1 h. Endogenous peroxidase was subsequently blocked by incubation with 3% H_2_O_2_ for 10–15 min, followed by two additional blocking steps with 0.001% avidin for 15 min and 0.001% biotin for 15 min, with several wash steps in between. This was followed by overnight incubation at 4°C with primary antibodies diluted in 5% goat serum at the following concentrations: anti-CLU (Proteintech Group Cat# 12289-1-AP, RRID : AB_2083316) 1 μg ml^-1^ and anti-Kim-1 (USBiological, Cat# 144807) 0.5-1 μg ml^-1^. After three wash steps, the biotinylated secondary antibody (Abcam Cat# ab97047, RRID : AB_10681025), diluted 1:200 in PBS for 1 h at room temperature was added, and subsequently washed with PBS. Enzyme activity was visualized using 3,3’-diaminobenzidine (DAB) (Abcam, Cat# ab64238) following 30 min incubation with the avidin and biotinylated horseradish peroxidase complex. Tissues were counterstained with hematoxylin, dehydrated, and mounted in Eukitt mounting medium (Sigma-Aldrich, Cat# 25608-33-7). Thresholds were set to detect DAB positivity, and the kidney sections were analyzed to determine the proportion of positive pixels for each biomarker. IHC profiler (Varghese et al., 2014) was used to assign an automated score to the respective IHC images by computerized pixel profiling based on colour deconvolution acquired on Image J developed at the National Institutes of Health (NIH, RRID: SCR_003070). The IHC optical density score for each zone is assigned as follows: 4 for the high positive zone, 3 for the positive zone, 2 for the low positive zone, and 1 for the negative zone. One-sided, two-sample t-tests assuming unequal variances were performed at the 5% significance level to test for variations in Kim-1 and CLU levels.

### Statistical Analyses

Statistical significance was determined by two‐way ANOVA with *post hoc* Dunnett’s test or Student’s t‐test as indicated. Statistical significance of the data was analyzed using Prism 8 software (GraphPad software, RRID: SCR_002798). Differences were deemed statistically significant if *P < 0.05 is defined as threshold, **P < 0.01 and ***P < 0.001. Data are presented as mean ± SEM. No statistical analysis was undertaken if a dataset had a group size (n) < 5.

## Results

### Comparative *In Vitro* Nephrotoxicity of SM21

#### Cell Viability by the CellTiter-Glo^®^ Assay in HK-2 Cells

**Figure 1:**
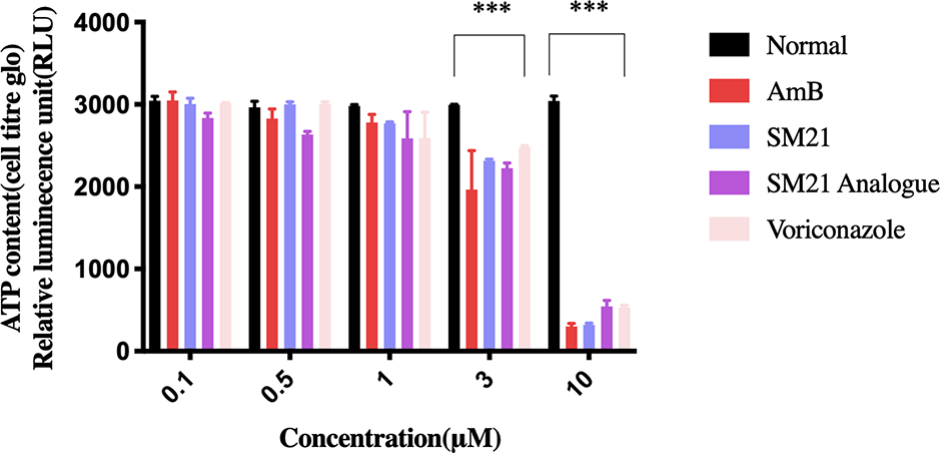
Evaluation of ATP metabolism. Concentration-response curve of cell viability in immortalized human proximal tubule epithelial cell line (HK-2) measured by the CellTiter-Glo® Luminescent Cell Viability assay. Trials were performed in triplicate from independently five times by use of different cell passages; non-treated cells with PBS in 1% DMSO used as vehicle. Each drug demonstrated dose-dependent depletion in ATP luminescence (significant difference observed from 1–10 μM). Significantly difference deemed as ***P < 0.001 relative to vehicle.

**Figure 2:**
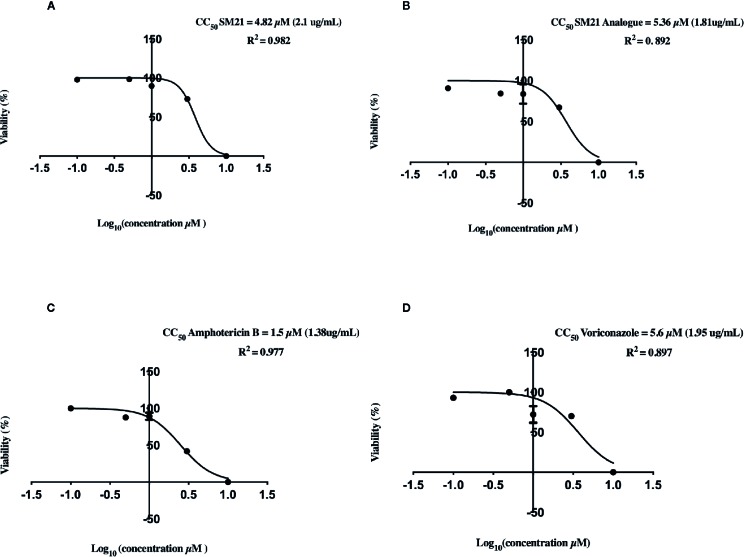
Cytotoxicity of each drug on HK-2 cells determined by quantitative CellTiter-Glo® of ATP Luminescent Cell Viability Assay. CC_50_ of Logarithm curves **(A)** SM21, **(B)** SM21 Analogue, **(C)** Amphotericin B, and **(D)** Voriconazole. The graphs were plotted based on logarithm of drug dosage and the CC_50_ was calculated by probit regression method using Prism 8. SM21 and its analogue showed lesser cytotoxicity and potency to induce proximal tubular damage in HK-2 compared to AmB (4.82, 5.36, and 1.5 μM; respectively). HK-2, immortalized human kidney proximal tubule epithelial cells.

Evaluation of cell viability by the CellTiter-Glo^®^ assay demonstrated dose-dependent (0.1, 0.5, 1, 3, and 10 μM) depletion in ATP luminescence for all drugs, with significant changes observed between 1 μM and 10 μM (**Figure 1**). AmB showed the highest potency to induce proximal tubular damage in HK-2 with a CC_50_ of 1.5 μM (1.38 μg ml^-1^). SM21 and SM21 analogue showed lesser cytotoxicity [CC_50_ 4.82 μM (2.1 μg ml^-1^) and 5.36 μM (1.81 μg ml^-1^), respectively] compared with AmB (**Figure 2**).

#### Antifungal Induced Expression of *CASP3* and *HAVcr-1* in HK-2 Cells

**Figure 3:**
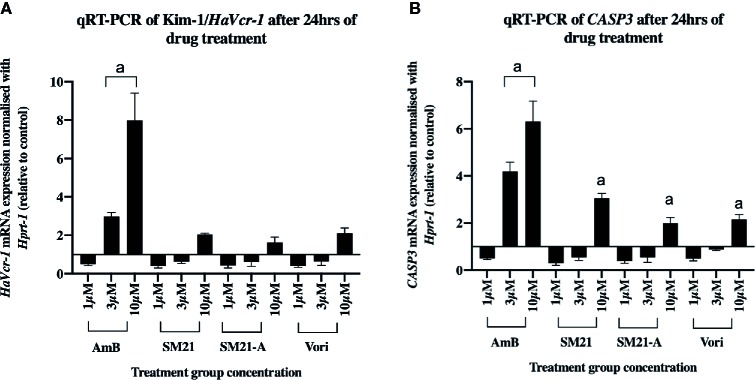
mRNA levels of *HaVcr-1*/Kim-1 and *CASP3*/Caspase-3 biomarkers in HK-2 cells after each compound treatment for 24 h. **(A)** *HaVcr-1* mRNA and **(B)** *CASP3* mRNA. Data are presented as mean standard deviation. Significantly difference ***P < 0.001 is depicted as "a" in the graphs, n>3 relative to control value as 1 (three independent assays with two technical replicates in each assay). Relatively higher gene expression of *Havcr1, CASP3* at lower concentration (3μM) in AmB was observed compared to SM21 treatment groups (SM21 and its analogue) and voriconazole. HK-2, immortalized proximal tubule epithelial cell; AmB, Amphotericin B; SM21-A, SM21 Analogue; Vori, Voriconazole.

To obtain further insights into the cell damage caused by each antifungal, real-time qPCR assay was used to determine mRNA levels of *HAVcr-1* and *CASP3* biomarkers of HK-2 cells after 24 h treatment with each drug (**Figure 3**). Upregulation of *HAVcr-1* and *CASP3* genes were observed at lower treatment concentrations for AmB and compared with the SM21 treatment group (SM21 and its analogue) and voriconazole. AmB induced significantly higher gene expression of *HAVcr-1* and *CASP3* compared with SM21 treatment groups at the concentration of 3 μM (**Figures 3A, B**, P< 0.001). Interestingly, the upregulation of these two genes was not observed until higher concentration (10 μM) in the SM21 treatment and voriconazole groups.

### *In Vivo* Nephrotoxicity of Antifungal Agents

#### General Signs and Symptoms of Animals

**Figure 4:**
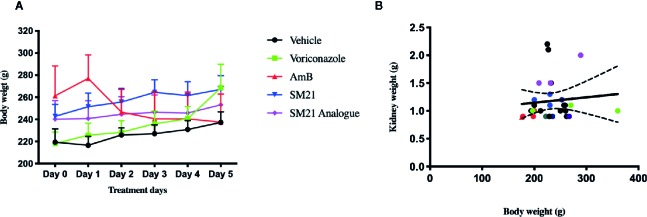
Change in kidney and body weights of male Sprague Dawley rats, **(A)** Mean body weight change in each group at 15 mg/kg/day BW over treatment period of 5 days. There were no clinical signs of toxicity at any time point and decrease in group mean body weights were observed in SM21 and analogue treatment groups in parallel with vehicle and negative control (Voriconazole) from day 1 to day 5. Whereas in Amphotericin B group, two of nine rats were removed on day 3 due to overt sign of toxicity at 15 mg/kg and slight decrease in body weight gain (–8%) compared with vehicle was observed in the rest of rats from day 1 to day 5. **(B)** Correlation between kidney weight and body weight of rats in each group on day 5. Kidney weights data were subjected to the linear regression and correlation was established with corresponding body weights using Prism's correlation matrix. There is poor or no correlation was found of kidney weights with body weights in the present study (P > 0.05).

No signs of toxicity were observed at any time point in the SM21 and SM21 analogue treated animal groups at a daily dose of 15 mg kg^-1^ BW (high dose), similar to that of the vehicle control and negative control (voriconazole) groups from day 1 to day 5. The animals in the preceding groups showed an increase in their mean body weights. On the contrary, animals treated with the positive control AmB at a daily dose of 15 mg kg^-1^ BW showed a slight decrease in body weight gain by day 5 compared with the control group (0.81 ± 0.07 mg kg-1 BW vs. 0.93 ± 0.06 mg kg-1 BW; respectively) (**Figure 4A**). Two animals treated with high dose AmB were removed from the study on day 3 due to overt signs of toxicity. However, the kidney weights of male SD rats in the present study did not show consistent pattern of relationship with terminal body weights (P > 0.05) (**Figure 4B**).

At a low dose (3 mg kg^-1^ BW/day), there were no overt signs of toxicity observed in the surviving rats across all treatment groups. Further, there was no apparent toxicity in kidney histopathology and no significant influence on body weight and clinical signs of toxicity during the observation period for the low dose of all antifungals. Therefore, only high dose groups were considered for further evaluation.

#### Serum Clinical Chemistry

On day 5, no statistically significant changes in serum BUN and SCr levels were noticed in SM21 and SM21 analogue treated rats compared with the vehicle control-treated rats. In the AmB treated group, a small but statistically significant increase of 1.4-fold and 2-fold was observed in SCr and BUN levels compared with the vehicle control group (**Table 1**).

**Table 1:** SCr and BUN were significantly increased at day 5 observed in AmB treated group whereas change of alterations of tested drug SM21 and its analogue treated groups fall in the range of negative control (voriconazole) and vehicle treated groups, but the magnitude of these alterations were much lower than that of almost all of the urinary kidney injury biomarkers (2-fold, 1.4-fold increases over vehicle-treated rats, respectively).

Group	Dose(mg/kg)	n	SCr(mg/dl)	BUN(mg/dl)
Vehicle	15	9	0.697 ± 0.1399	4.386 ± 0.917
Voriconazole	15	9	0.717 ± 0.154	4.140 ± 0.790
AmB	15	7	**1.044 ±0.119	*6.35 ± 0.591
SM21	15	9	0.801 ± 0.075	4.87 ± 1.308
SM21 Analogue	15	9	0.801 ± 0.069	4.96 ± 1.529

Significantly difference deemed as *P < 0.05; **P< 0.01. SCr, Serum creatinine; BUN, Blood urea nitrogen; AmB, Amphotericin B.

#### Urine Clinical Chemistry and Novel Kidney Injury Biomarkers

**Figure 5:**
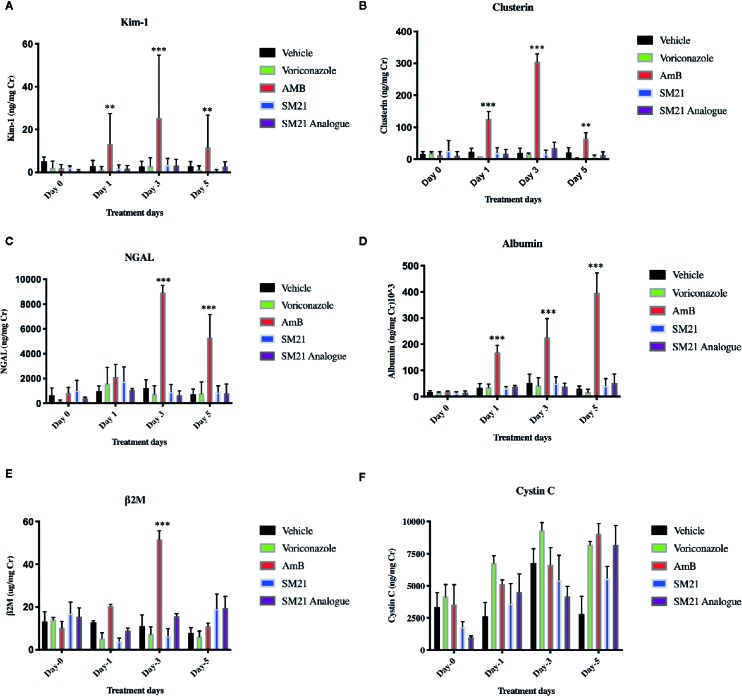
Time course for the appearance of next generation urinary biomarkers . The urinary level of the biomarkers **(A)** Kim-1, **(B)** Clusterin , **(C)** NGAL **(D)** Albumin, **(E)** β2M, **(F)** Cystin C before and after administration of AmB, Voriconazole, SM21 and SM21 Analogue in male SD rats at dose of 15 mg/kg/BW compared with vehicle-treated controls [n =9 for each treatment group except AmB group (n=7)]. Data are expressed as means ± SD. The statistical significance of differences between treated and control groups was determined using a repeated measures analysis, **p < .01, and ***p < .001 at each sampling point by ANOVA and Dunnett's test. The quantity of biomarkers in urine was presented after normalization, based on the concentration of urinary creatinine. Significant expression of Kim-1, NGAL, Clusterin, and Albumin were observed with AmB at day 1 & 3. In contrast, the expression of these biomarkers in SM21 and its analogue were not statistically significant compared to vehicle (saline) and voriconazole groups. Kim-1, Kidney injury molecule-1; NGAL, Neutrophil gelatinase-associated lipocalin; β2M, β macroglobulin; AmB, Amphotericin B.

Rats treated with AmB at 15 mg kg^-1^ BW/day showed statistically significant upregulation of urinary Kim-1, CLU, ALB, and NGAL compared with the vehicle control group (**Figures 5A–E**). In contrast, rats treated with SM21 and SM21 analogue at 15 mg kg^-1^ BW/day did not show any statistically significant increase in, Kim-1, CLU, ALB, and NGAL levels compared with the vehicle and negative control voriconazole groups (**Figures 5A–D**; respectively). In AmB group, urinary concentrations of Kim-1, CLU and NGAL, β2M started to increase as early as day 1, with a statistically significant increase observed by day 3 whereas ALB remain increasing up to day 5 (**Figures 5A–E**; respectively). On day 3 post-treatment, significant elevations were noted within the AmB group in urinary concentrations of Kim-1, CLU, NGAL, and ALB (approximately 6-, 15-, 5-, and 4- fold increases, respectively, compared with vehicle-treated rats, P < .001).

#### Histopathology of Kidneys

**Figure 6:**
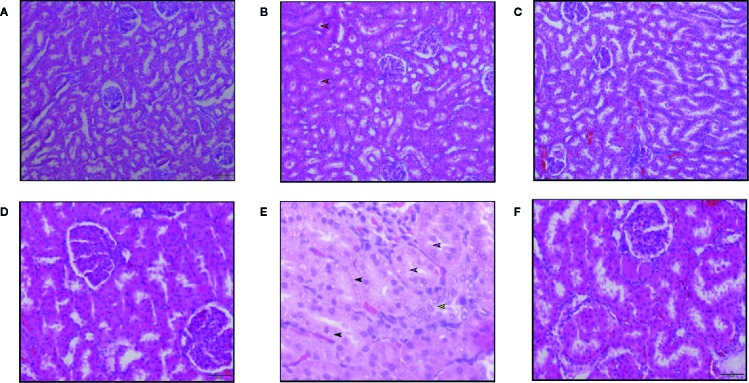
Hematoxylin and eosin staining of kidney sections of Sprague Dawley rats treated with 15 mg/kg/day BW of Vehicle, SM21 and Amphotericin B groups after treatment for 5 days. Six micrographs taken from the cortex of kidney in Vehicle **(A, D)**, Amphotericin B **(B, E)**, SM21 **(C, F)** groups. Control groups [Vehicle and voriconazole (not shown)] showed no abnormality. SM21 treatment group (SM21 and its analogue) had similar findings of the control groups along with mild tubular necrosis. Among tubular necrosis in Amphotericin B group, loss of the brush border of tubuli (pink arrow in Figure **E**), mild tubular cell vacuolation (black arrow in Figure **E**), intratubular cast formation (red arrow in Figure **B**), disengagement of cells into lumen of tubuli (yellow arrow in Figure **E**) were significantly observed. SD, Sprague Dawley.

H&E sections of the kidneys obtained from rats treated with high dose AmB (15 mg kg^-1^ BW/day) showed features of necrosis. Loss of proximal tubule lining cells was graded primarily as mild to moderate and as more extensive proximal tubule necrosis. Loss of the brush border of tubuli (**Figure 6E**), moderate tubular cell vacuolation (**Figure 6E**), intratubular cast formation (**Figure 6B**), disengagement of cells into the lumen of tubuli (**Figure 6E**) were significantly observed in AmB treated group, but there was no evidence of severe glomerular damage. On the contrary, there were no major tubular epithelial abnormalities observed in the histopathology sections of kidneys obtained from rats treated with SM21 (**Figures 6C, F**) and it’s analogue and negative control voriconazole animals at any time point with similar dose of 15 mg kg^-1^ BW/day).

#### Correlation Between Urine Clinical Chemistry and Kidney Histopathology

**Figure 7:**
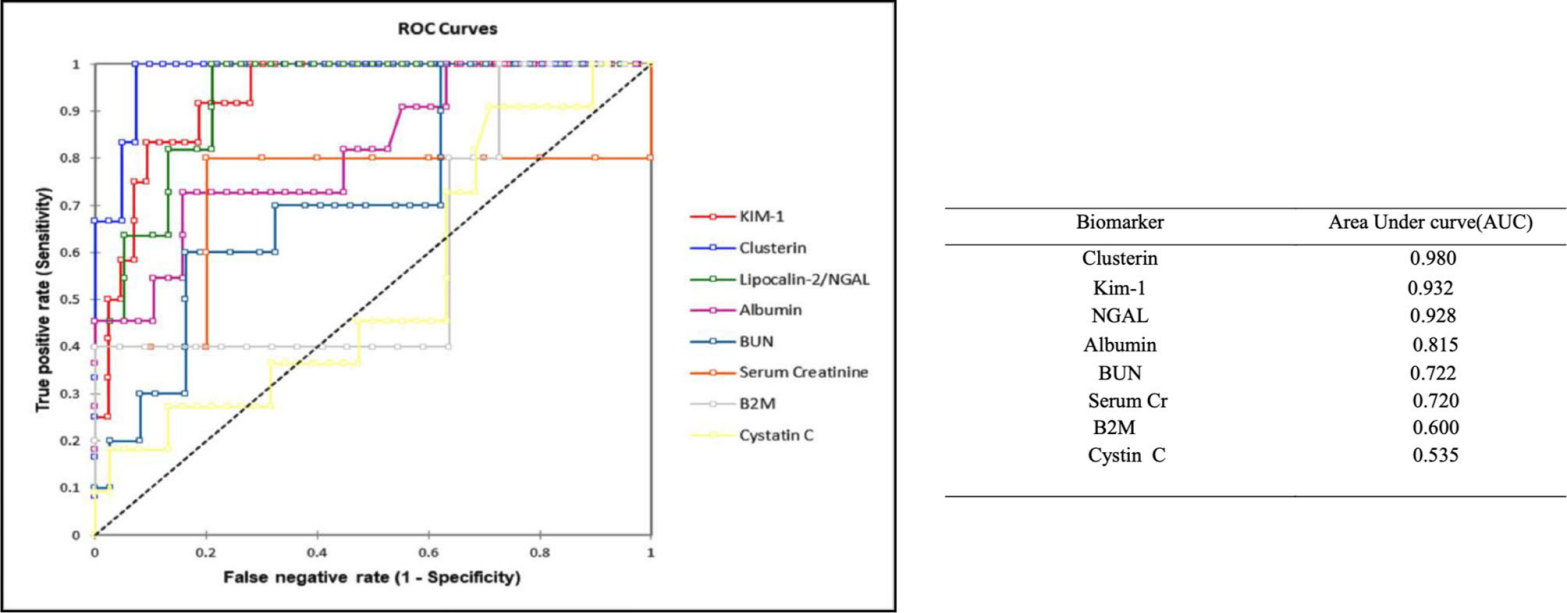
Receiver-operator characteristics (ROC) curves for next generation urinary biomarkers compared to traditional clinical chemistry parameters. The area under the ROC curve, which serves as measure for the overall ability of a biomarker to discriminate normal (control) vs. diseased AmB and SM21 treatment (SM21 and its analogue) animals, is given in parenthesis. The pathological lesions were graded from 0–3 to calculate ROC. We defined a ROC-AUC of 0.5–0.6 as showing no predictive ability, a ROC-AUC of 0.6–0.7 as showing poor predictive ability, a ROC-AUC of 0.7–0.8 as showing fair predictive ability, a ROC-AUC of 0.8–0.9 as showing good predictive ability, and a ROC-AUC above 0.9 as showing outstanding predictive ability. An excellent diagnostic performance in our study for clusterin and Kim-1 (AUC- 0.98 and 0.932, respectively) to evaluate comparative nephrotoxicity of AmB compared to control groups and SM21 treatment group [These binomially plotted ROC curves were created using XLSTAT software (Addinsoft, Microsoft)]. Kim-1, Kidney injury molecule-1; NGAL, Neutrophil gelatinase-associated lipocalin; β2M, β macroglobulin; AmB, Amphotericin B.

The novel urinary biomarkers and classical biomarkers of SCr and BUN were compared with the renal tubular alterations observed in the kidney histopathology. The pathological lesions were graded from 0 to 3 to calculate receiver operation-characteristic curves (ROC). The investigated area-under-the-ROC (AUROC) served as a measure of the overall ability to discriminate normal (vehicle) vs. antifungal treated animals (**Figure 7**). AUROC values > 0.9 were considered as good biomarkers with satisfactory diagnostic performance. However, biomarkers with AUROC between 0.7 and 0.9 can also be of diagnostic value. These bionomally plotted ROC curves were created using XLSTAT software version 2019.1 (Adinosft, Microsft). An excellent diagnostic performance was observed in our study for CLU and Kim-1 (AUC 0.98 and 0.932, respectively) for evaluating comparative nephrotoxicity of AMB compared with the control (vehicle and negative control voriconazole) and SM21 treatment groups (SM21 and the analogue). Hence, to obtain more evidence on this interesting observation, immunohistochemical analysis was performed for localization of these two proteins in the kidney tissues of each group.

ALB and NGAL levels were also increased in several animals in the AmB group, with AUROC values of 0.928 and 0.815, respectively, underlying the diagnostic performance of these two markers. In contrast, β2M (AUC-0.6) was less sensitive than SCr (AUC-0.72) and BUN (AUC-0.722), while Cystin C (AUC-0.535) was the least responsive marker of renal injury in the present study.

#### Localization of Kim-1 and Clusterin Protein in Renal Tissue by Immunohistochemical Analysis

**Figure 8:**
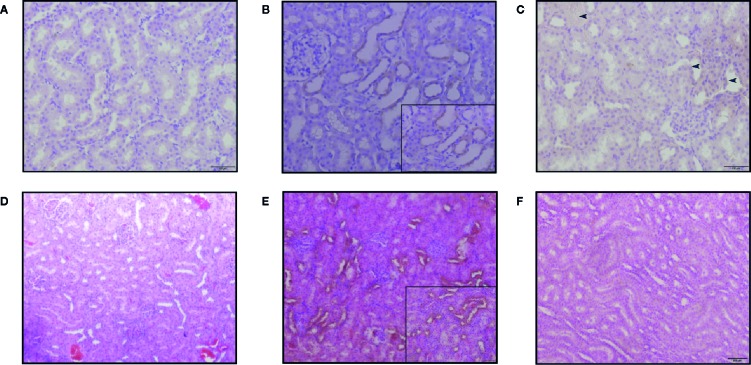
Kim-1 and CLU immunoreactivity in the kidneys of SD rats treated with a single intravenous dose of 15mg/kg/day BW for 5 days. In Kim-1 immunoreactivity was shown in, **(A)** vehicle, **(B)** AmB and **(C)** SM21. Only occasional Kim-1-positive tubules were detected in SM21 (blue arrow in C) kidneys. Kim-1 was prominently detected translocated to an apical (luminal) membrane of convoluted portion of the proximal tubules in AmB group. In CLU immunoreactivity was shown in, **(D)** Vehicle **(E)** AmB, **(F)** SM21. In AmB group, high expression of CLU observed over almost all different tubules. Positive pixel analysis identified a low increase in immunoreactivity in rats dosed with SM21 group compared to control. However, this expression is comparatively lower than AmB treatment. No immunoreactivity of Kim-1 and CLU were detected in vehicle group. IHC profiler was used to assign an automated score to the respective IHC images by computerized pixel profiling based on colour deconvolution acquired on Image J. Kim-1, Kidney injury molecule-1; CLU, Clusterin; SD, Sprague Dawley; IHC, Immunohistochemistry.

Kim-1 and CLU showed no staining in most control animals (**Figure 8A**) and the voriconazole group, while minimal expression within the cortical tissue of the kidney was observed in the SM21 treatment groups (**Figure 8C**). In contrast, it was clearly observed that Kim-1 translocated to an apical (luminal) membranous and/or cytoplasmic (sub-membranous) distribution in the AmB high dose group (**Figure 8B**). This distinctive pattern was observed in the straight portion of the proximal tubules and then extended to the convoluted portion of the proximal tubules, where the morphological degenerative changes were more pronounced. On the contrary, none to a minimal amount of intraluminal Kim-1 protein was observed at the apical membrane of proximal tubular cells within the cortex in the SM21 treatment group (**Figure 8C**). Kim-1 immunoreactivity had been reported to remain detectable in a few tubules up to at least 14 days after exposure to the nephrotoxicant (Ichimura et al., 2004). As reported, The Kim-1 localization on day 5 consistent with our results, wherein necrotized kidneys of AmB treated rats, the staining pattern is predominantly apical. In agreement with other groups (de Borst et al., 2006; Vinken et al., 2012), we detected low levels of Kim-1 immunoreactivity in some control rat kidneys and SM21 treatment groups (**Figures 8A, C**), likely indicating low levels of naturally occurring tubular injury/regeneration.

High CLU expression was observed over almost all different tubules in the AmB group (**Figure 8E**). The specificity of CLU localization was weaker than that of Kim-1 because the location of the kidney injury was not linked to any specific tubular segment. CLU immunoreactivity was absent in control animals at all time points. Positive pixel analysis identified a low increase in immunoreactivity in rats in the SM21 treatment group compared with control (**Figure 8F**). However, this expression was comparatively lower than the AmB group. No immunoreactivity of Kim-1 and CLU was detected in any of the goat IgG isotype negative controls at any time point.

**Figure 9:**
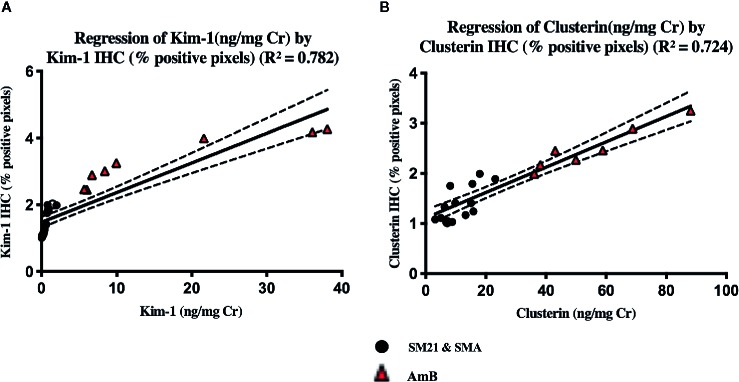
There was a significant level of correlation between tissue (x-axis) and urinary (y-axis), **(A)** Kim-1 and **(B)** Clusterin from individual animals on the day 5. Vehicle, SM21 and SM21 analogue treated rats had little to no urinary expression of protein nor protein immunoreactivity within the kidney, whereas AmB-treated rats had higher levels of both urinary protein expression and immunoreactivity observed within the kidney. The line of best following linear regression of Kim-1 and clusterin are shown (R^2^ = 0.782) and (R^2^ = 0.724), respectively. The IHC optical density score calculation (Image J) of the each zone is assigned as 4 for the high positive zone, 3 for the positive zone, 2 for the low positive zone, and 1 for the negative zone. IHC, immunohistochemistry; Kim-1, Kidney Injury molecule-1; SMA, SM21 Analogue.

To determine whether these tissue biomarkers can provide the same information as urinary biomarkers, the normalized values of urinary Kim-1 and CLU on day 5 were correlated with the respective IHC data for AmB and SM21 treatment animals. **Figure 9** shows the relationships between the tissue and urinary biomarker data, using the urine collected on the same day the animals were terminated (day 5). The IHC data showed a positive correlation with the urine clinical chemistry data for both Kim-1 and CLU (R^2^.784 and.724; respectively).

## Discussion

The present study aimed to comparatively evaluate the nephrotoxicity of the novel antifungal small molecule SM21 with existing antifungals of AmB and voriconazole under both *in vitro* and *in vivo* conditions. In this comprehensive study, we used both traditional and novel nephrotoxicity markers for this purpose. The study results demonstrate that SM21 has minimal potential to cause acute nephrotoxicity compared with AmB, a widely used broad-spectrum antifungal agent. SM21 treatment causes minimal renal tubule necrosis and no major tubular epithelial abnormalities observed at a single dose of 15 mg kg^-1^ BW/day. At this similar dose of AmB treatment showed pronounced necrosis in renal epithelial tubular cells (85% of the tubules). The results are consistent with the previous reported study by Varlam et al., 2001.

Organ weight is one of the most sensitive drug toxicity indicators, and its changes often precede morphological changes (Piao et al., 2013). Previous studies have suggested that kidney weight of male rats could be optimally analyzed with the body weight drawn through covariance statistical models (Bailey et al., 2004; Nirogi et al., 2014). Although statistical models have been widely used in the evaluation of organ weights in toxicology studies, their reliability and implications have been challenged by several studies (Gad and Rousseaux, 2002). In the present study, no significant correlation was found in kidney weight with body weight of male SD rats (P > 0.05). Nevertheless, all animals concomitantly treated with SM21 drugs and voriconazole drug gained more weight than rats treated with AmB alone. Further study is warranted to investigate the long-term renal toxicity of novel drug SM21 treatment.

Interestingly, histopathological observation of kidney injury in the AmB group did not correlate well with SCr and BUN levels, which are regarded as the current gold standard biomarkers for kidney toxicity. Therefore, they may not be sensitive indicators of structural injury, in part because of the excess capacity—or “renal reserve”—of the kidneys as reported (Ferguson et al., 2008; Parikh and Devarajan, 2008). Hence, a panel of promising novel renal biomarkers, which have been suggested as early indicators of nephrotoxicity, were used to evaluate nephrotoxicity (Sistare and DeGeorge, 2007). Our results show that the activity of several novel urinary nephrotoxicity biomarkers, *i.e.,* Kim-1, CLU, NGAL, and β2M are significantly augmented in a time-dependent manner up to day 3 and ALB up to day 5 in the AmB group compared with the SM21 and control groups. The high AUC values from ROC curves observed for CLU and Kim-1 indicate that these biomarkers are the most sensitive in discriminating between nephrotoxicity observed in the AmB-treated animals and the other lesser-nephrotoxic groups. The significant increase in the urinary levels of these two proteins correlates well with their immunolocalization at the site of injury, involving proximal convoluted tubules (PCT), and to a lesser extent, distal convoluted tubules (DCT) in kidneys.

Our observation that Kim-1 is significantly more sensitive than the traditional clinical chemistry parameters for kidney injury is consistent with that of previous studies. We observed that urinary Kim-1 protein levels *in vivo* is very sensitive to the renal injury produced by AmB. This further corroborates the safety aspect of SM21 compared with the highly nephrotoxic AmB and confirms that urinary Kim-1 could be considered as sensitive biomarker for early detection of nephrotoxicant-induced proximal tubular injury. For instance, increased levels of urinary Kim-1 levels were reported correlate well with the degree of kidney injury and Kim-1 gene expression in kidney in some studies (Ichimura et al., 2008; Rached et al., 2008; Zhou et al., 2008; Prozialeck et al., 2009). The comprehensive review by Chen et al., 2018 indicated that increased usage of Kim-1 as a biomarker for detection of kidney injury in drug development programs reviewed by the Center for Drug Evaluation and Research (CDER) within past 10 years. Moreover, Kim-1 was found to be highly detectable at the site of PCT in injured renal tissue of humans and rodents, irrespective of the mode of renal injury (Tonomura et al., 2010; Vaidya et al., 2010b; Yang et al., 2015).

Similar to Kim-1 excretion, CLU, another novel nephrotoxic biomarker attained the highest ROC value in our study, suggesting that it is a good biomarker for AmB-induced nephrotoxicity. One of the major advantages of using CLU as a kidney injury biomarker is that it is secreted directly into the urine after renal insult. Even though Urinary clusterin detects tubular injury, with similar performance and characteristics to Kim-1, some studies have reported the contradictory view on the sensitivity and localization of CLU, as it may be dependent on the nature of the nephrotoxicant (Sieber et al., 2009; Wadey et al., 2014).

The epithelial cells of the renal proximal tubule (PTC) are a major target for toxic drug effects in the kidney. We used human origin HK-2 kidney proximal tubular epithelial cell lines. HK-2 cells are PTC-derived and were established in 1994 by immortalization with human papilloma virus-16 (HPV-16) E6/E7 genes (Ryan et al., 1994). It has shown high vulnerability to mitochondrial dysfunction in acute renal injury (Gunness et al., 2011). Further, the cells were shown to express various PTC-specific brush border enzymes and epithelial markers, and they display functional characteristics of PTC (Ryan et al., 1994). Renal drug transporters play an important role in the absorption, elimination, metabolism, and toxicity of many prescribed drugs. The basolateral membrane transporters include both the organic anion (e.g. OAT1, OAT3, OATP4C1) and the organic cation transporter families (e.g. OCT2), which mediate the uptake of anionic and cationic drugs from the blood into proximal tubule cells. Apical membrane transporters function both to secrete drugs into the urine and to reabsorb compounds from the urine back into the proximal tubule cells. These transporters include the multidrug and toxin extrusion proteins (MATE1, MATE2-K), the multidrug resistance protein 1 (MDR1, P-glycoprotein, or P-gp), multidrug resistance-associated proteins (MRP2, MRP4), the oligopeptide transporters (PEPT1, PEPT2), additional organic anion and cation transporters (OAT4, OCTN1, OCTN2) and a urate transporter (URAT1). Recently it was demonstrated that HK-2 cells express the monocarboxylate transporter 1 (MCT1), the basolateral transporter OATP4C1 and with respect to the apical membrane efflux transporters ABCB1 (MDR1) and multidrug resistance proteins (MRPs). However, expression of the major basolateral organic anion (OAT) uptake transporters OAT1 and OAT3 and of the organic cation (OCT) uptake transporter OCT2 could not be detected (Jenkinson et al., 2012). Therefore, it is important to validate the findings observed in HK-2 cells using primary kidney cells like RPTEC/TERT1 and SA7K human kidney proximal tubule cell lines in future studies.

Due to discrepancies in the literature about the use of MTT assay as an initial screening test to establish drug-induced cell viability (Stepanenko and Dmitrenko, 2015), we used the ATP luminescent assay. ATP (adenosine triphosphate) represents the most important chemical energy reservoir in cells and is used for biological synthesis, signaling, transport, and movement processes. When cells damaged lethally and lose membrane integrity, they lose the ability to synthetize ATP and the ATP level of cells decreases dramatically (García and Massieu, 2003). The ATP detection assay is by far the most sensitive end points in measuring cell viability and is less prone to artefacts than other viability assays (Riss et al., 2004). There is a linear relationship between the intensity of luminescent signal and ATP concentration (Mueller et al., 2004) or cell number (Andreotti et al., 1995). ATP values higher than controls (untreated cells) has been indicated as proliferation and cultures with ATP concentrations lower than controls indicate cytotoxicity (BioAssay Ontology-EMBL-EBI, 2019). This assay has been particularly useful because it is sensitive enough to reproducibly detect the ATP production from a single mammalian cell (Paciello et al., 2010). However, further consideration should be given for performing additional cytotoxicity and/or cell viability assays to assure the reproducibility of our current data.

Cell death due to nephrotoxic injury has always been thought to result through necrosis. Recently, apoptosis has been identified as an alternate mechanism of nephrotoxic cell death, as it is a very common event in compound-induced toxicity (Havasi and Borkan, 2011). This is especially true in the proximal tubules, which have a high abundance of mitochondria to facilitate their transport and secretory functions. The mitochondria are also a key site for mediating cell death *via* the release of proapoptotic inducers and the production of ATP, a master regulator of apoptosis (Linkermann et al., 2014). The mitochondrial-dependent pathway involves the release of cytochrome c and Smac/Diablo regulates the activation of caspase-9 and subsequently, caspase-3.

*CASP3* and Kim-1/*HAVcr-1* genes as biomarkers in detecting in-vitro toxicity induced by AmB were proposed by Grossi et al., 2017. Our results are consistent with the foregoing study showing the over-expression of genes of *HAVcr-1* and *CASP3* in response to AmB treatment as indicated by mRNA profiling. In contrast, we observed a downregulation of *CASP3* and *HAVcr-1* expression in the treatment group with 3µM of SM21 in HK-2 cells. The down-regulation of the genes *CASP3* and *HAVcr-1* from SM21 treatment suggest that this drug has lower nephrotoxicity than AmB. This observation can be further corroborated in future studies by examining the protein expression levels of *CASP3* and *HAVcr-1* at 24 h or later post-treatment.

## Conclusion

In conclusion, the present study demonstrated that SM21 has a significantly better safety profile in terms of nephrotoxicity with no major tubular epithelial abnormalities observed in kidney cells and no augmentation of kidney injury biomarker levels compared with AmB. The novel antifungal small molecule SM21, therefore, holds significant promise and should be further examined for other possible side effects before proceeding to the clinical trials. Moreover, our study also found that the urinary CLU and Kim-1 level are the most sensitive biomarkers that can accurately predict antifungal induced acute kidney injury earlier than the traditional biomarkers serum SCr and BUN. The future clinical trials should include urinary CLU and Kim-1 as early indicators of acute kidney injury in antifungal induced nephrotoxicity.

## Data Availability Statement

The datasets generated for this study are available on request to the corresponding authors.

## Ethics Statement

The animal study was reviewed and approved by Institutional Animal Care and Use Committee, Singapore.

## Author Contributions

NU, SK, TA, and CS participated in the research design. NU, SK, and TA conducted the experiments. NU performed the data analysis. NU, SK, TA, YW and CS wrote or contributed to the writing of the manuscript. All authors reviewed and revised the final version of manuscript and approved manuscript submission.

## Funding

This work was fully funded by National Medical Research Council, Singapore (NMRC/CIRG/1455/2016) to CS.

## Conflict of Interest

The authors declare that the research was conducted in the absence of any commercial or financial relationships that could be construed as a potential conflict of interest.

## Abbreviations

ALB, Albumin; β2M, Beta-2 microglobulin; BUN, Blood urea nitrogen; *CASP3*, Casapase 3 gene; CLU, Clusterin; Cys C, Cystin C; *HAVcr-1*, Hepatitis A virus cellular receptor 1 gene; Kim-1, Kidney injury molecule-1; NGAL, Neutrophil gelatinase-associated lipocalin; SCr, Serum Creatinine; BUN, Blood urea nitrogen.

## References

[B1] Adedeji A. O. Pourmohamad T. Chen Y. Burkey J. Betts C. J. Bickerton S. J. (2019). Investigating the Value of Urine Volume, Creatinine, and Cystatin C for Urinary Biomarkers Normalization for Drug Development Studies. Int. J. Toxicol. 38 (1), 12–22. 10.1177/1091581818819791 30673360

[B2] Andreotti P. E. Cree I. A. Kurbacher C. M. Hartmann D. M. Linder D. Harel G. (1995). Chemosensitivity Testing of Human Tumors Using a Microplate Adenosine Triphosphate Luminescence Assay: Clinical Correlation for Cisplatin Resistance of Ovarian Carcinoma. Cancer Res. 55 (22), 5276–5282. 7585588

[B3] Bailey S. A. Zidell R. H. Perry R. W. (2004). Relationships Between Organ Weight and Body/Brain Weight in the Rat: WhaT Is the Best Analytical Endpoint? Toxicol. Pathol. 32 (4), 448–466. 10.1080/01926230490465874 15204968

[B4] *BioAssay Ontology-EMBL-EBI* (2019). Available at: https://www.ebi.ac.uk/ols/ontologies/bao/terms?iri=http://www.bioassayontology.org/bao%23BAO_0000187.

[B5] Chen R. Sanyal S. Thompson A. Ix J. H. Haskins K. Muldowney L. (2018). Evaluating the Use of KIM-1 in Drug Development and Research Following FDA Qualification. Clin. Pharmacol. Ther. 104 (6), 1175–1181. 10.1002/cpt.1093 29761868 PMC6226328

[B6] Dantas Ada S. Day A. Ikeh M. Kos I. Achan B. Quinn J. (2015). Oxidative stress responses in the human fungal pathogen, Candida albicans. Biomolecules 5 (1), 142–165. 10.3390/biom5010142 25723552 PMC4384116

[B7] de Borst M. H. Timmeren M. M. Vaidya V. S. de Boer R. A. van Dalen M. B. Kramer A. B. (2006). Induction of kidney injury molecule-1 in homozygous Ren2 rats is attenuated by blockade of the renin-angiotensin system or p38 MAP kinase. Am. J. Physiol-Renal Physiol., F313–F320. 10.1152/ajprenal.00180.2006 16896183

[B8] Dieterle F. Sistare F. Goodsaid F. Papaluca M. Ozer J. S. Webb C. P., (2010). Renal biomarker qualification submission: A dialog between the FDA-EMEA and Predictive Safety Testing Consortium. Nat. Biotechnol. 28 (5), 455–462. 10.1038/nbt.1625 20458315

[B9] Felton T. Troke P. F. Hope W. W. (2014). Tissue penetration of antifungal agents. Clin. Microbiol. Rev. 27 (1), 68–88. 10.1128/CMR.00046-13 24396137 PMC3910906

[B10] Ferguson M. A. Vaidya V. S. Bonventre J. V. (2008). Biomarkers of nephrotoxic acute kidney injury. Toxicology 245 (3), 182–193. 10.1016/j.tox.2007.12.024 18294749 PMC4038970

[B11] Fuchs T. C. Hewitt P. (2011). Biomarkers for Drug-Induced Renal Damage and Nephrotoxicity—An Overview for Applied Toxicology. AAPS J. 13 (4), 615–631. 10.1208/s12248-011-9301-x 21969220 PMC3231866

[B12] Gad S. Rousseaux C. (2002). Handbook of Toxicologic Pathology. Edition 2 San Diego, California: Academic Press 327–418.

[B13] García O. Massieu L. (2003). Glutamate Uptake Inhibitor L-Trans-Pyrrolidine 2,4-Dicarboxylate Becomes Neurotoxic in the Presence of Subthreshold Concentrations of Mitochondrial Toxin 3-Nitropropionate: Involvement of Mitochondrial Reducing Activity and ATP Production. J. Neurosci. Res. 74 (6), 956–966. 10.1002/jnr.10825 14648602

[B14] Grossi M. F. Campos M. F. Soares M. A. A. Silva S. Nunes S. C. T. Almeida M. S. (2017). In Vitro Study of Potential Nephrotoxicity Biomarkers through Gene Expression Using Reverse sequence. J. Toxicol. Pharmacol. 1 (1), 0–4.

[B15] Gunness P. Aleksa K. Kosuge K. Ito S. Koren G. (2011). Corrigendum: Comparison of the novel HK-2 human renal proximal tubular cell line with the standard LLC-PK1 cell line in studying drug-induced nephrotoxicity. Can. J. Physiol. Pharmacol. 89 (10), 767–767. 10.1139/y11-078 20555413

[B16] Havasi A. Borkan S. C. (2011). Apoptosis and acute kidney injury. Kidney Int. 80 (1), 29–40. 10.1038/ki.2011.120 21562469 PMC4625984

[B17] Hill R. G. Rang H. P. (2012). Drug discovery and development : technology in transition. (Churchill Livingstone: Elsevier).

[B18] Ichimura T. Hung C. C. Yang S. A. Stevens J. L. Bonventre J. V. (2004). Kidney injury molecule-1: a tissue and urinary biomarker for nephrotoxicant-induced renal injury. Am. J. Physiol.-Renal Physiol. 286 (3), F552–F563. 10.1152/ajprenal.00285.2002 14600030

[B19] Ichimura T. Asseldonk E. J. Humphreys B. D. Gunaratnam L. Duffield J. S. Bonventre J. V., (2008). Kidney injury molecule-1 is a phosphatidylserine receptor that confers a phagocytic phenotype on epithelial cells. J. Clin. Invest. 118 (5), 1657–1668. 10.1172/JCI34487 18414680 PMC2293335

[B20] International Society of Nephrology (2012). “Chapter 5.4: Vascular acces for renal replacement therapy,” in AKI’, KDIGO Clinical Practice Guideline for Acute Kidney Injury, vol. 2 , 3. KDIGO® AKI Guideline Online Appendices A-F March 2012 10.1038/kisup.2012.3

[B21] Jenkinson S. E. Chung G. W. van Loon E. Bakar N. S. Dalzell A. M. Brown C. D. (2012). ‘The limitations of renal epithelial cell line HK-2 as a model of drug transporter expression and function in the proximal tubule. Pflügers Archiv : Eur. J. Physiol. 464 (6), 601–611. 10.1007/s00424-012-1163-2 23014881

[B22] Journal S. Nephrotoxicity A. B. (2002). of Kidney Diseases and Transplantation Review Article Amphotericin B Nephrotoxicity. Saudi J. Kidney Dis. Transpl. 13, 4, 481–491. 17660672

[B23] Keane S. Geoghegan P. Povoa P. Nseir S. Rodriguez A. Martin-Loeches I. (2018). Systematic review on the first line treatment of amphotericin B in critically ill adults with candidemia or invasive candidiasis. Expert Rev. Anti-Infective Ther. 16 (11), 839–847. 10.1080/14787210.2018.1528872 30257597

[B24] Kola I. Landis J. (2004). Can the pharmaceutical industry reduce attrition rates? Nat. Rev. Drug Discovery 3 (8), 711–716. 10.1038/nrd1470 15286737

[B25] Kuznar W. Baglin T. (2015). In Search of the Holy Grail of Anticoagulant Therapy. MD Conf. Express 13 (13), 12–13. 10.1177/155989771313005

[B26] Linkermann A. Chen G. Dong G. Kunzendorf U. Krautwald S. Dong Z. (2014). Regulated Cell Death in AKI. J. Am. Soc. Nephrol. 25 (12), 2689–2701. 10.1681/ASN.2014030262 24925726 PMC4243360

[B27] Livak K. J. Schmittgen T. D. (2001). Analysis of relative gene expression data using real-time quantitative PCR and the 2-ΔΔCT method. Methods 25 (4), 402–408. 10.1006/meth.2001.1262 11846609

[B28] Mueller H. Kassack M. U. Wiese M. (2004). Comparison of the usefulness of the MTT, ATP, and calcein assays to predict the potency of cytotoxic agents in various human cancer cell lines. J. Biomol. Screening 9 (6), 506–515. 10.1177/1087057104265386 15452337

[B29] Nirogi R. Goyal V. K. Jana S. Pandey S. K. Gothi A (2014). What Suits Best for Organ Weight Analysis : Review of Relationship Between Organ Weight and Body / Brain Weight for Rodent Toxicity Studies. Int. J. Pharmaceutical Sci. Res. 5 (4), 1525–1532. 10.13040/IJPSR.0975-8232.5(4).1525-32

[B30] Olson J. A. Adler-Moore J. P. Jensen G. M. Schwartz J. Dignani M. C. Proffitt R. T. (2008). Comparison of the physicochemical, antifungal, and toxic properties of two liposomal amphotericin B products. Antimicrobial Agents Chemother. 52 (1), 259–268. 10.1128/AAC.00870-07 PMC2223902 17967910

[B31] Ostrosky-Zeichner L. Casadevall A. Galgiani J. N. Odds F. C. Rex J. H. (2010). An insight into the antifungal pipeline: Selected new molecules and beyond. Nat. Rev. Drug Discovery 9 (9), 719–727. 10.1038/nrd3074 20725094

[B32] Paciello L. Falco F.C. Parascandola Palma (2010). Determination of yeast cell viability: Viable count vs ATP-based bioluminescence assay. J. Biotechnol. 150, 386–387. 10.1016/j.jbiotec.2010.09.488 23410926

[B33] Parikh C. R. Devarajan P. (2008). New biomarkers of acute kidney injury. Crit. Care Med. 36 (SUPPL. 4), 159–165. 10.1097/CCM.0b013e318168c652 18382188

[B34] Perlroth J. Choi B. Spellberg B. (2007). Nosocomial fungal infections: Epidemiology, diagnosis, and treatment. Med. Mycol. 45 (4), 321–346. 10.1080/13693780701218689 17510856

[B35] Pfaller M. A. (2012). Antifungal drug resistance: Mechanisms, epidemiology, and consequences for treatment. Am. J. Med. 125 (1 SUPPL.), S3–S13. 10.1016/j.amjmed.2011.11.001 22196207

[B36] Pharmacology of amphotericin B (2019). Available at: https://www.uptodate.com/contents/pharmacology-of-amphotericin-b.

[B37] Piao Y. Liu Y. Xie X. (2013). Change trends of organ weight background data in Sprague Dawley rats at different ages. J. Toxicol. Pathol. 26 pp (1), 29–34. 10.1293/tox.26.29 23723565 PMC3620211

[B38] Promega (2013). CellTiter Glo PROMEGA. Data Sheet 138 (1), 66–83. 10.1016/j.pharmthera.2013.01.002

[B39] Prozialeck W. C. Vaidya V. S. Liu J. Waalkes M. P. Edwards J. R. Lamar P. C. (2009). Kidney injury molecule-1 is an early biomarker of cadmium nephrotoxici. Kidney Int. 72 (8), 985–993. 10.1038/sj.ki.5002467.Kidney PMC2747605 17687258

[B40] Rached E. Hoffmann D. Blumbach K. Weber K. Dekant W. Malley A. (2008). Evaluation of putative biomarkers of nephrotoxicity after exposure to ochratoxin a in vivo and in vitro. Toxicol. Sci. 103 (2), 371–381. 10.1093/toxsci/kfn040 18308701

[B41] Riss T. L. Moravec R. A. Niles A. L. Duellman S. Benink H. A. Worzella T. J. (2004). Assay Guidance Manual [Internet]. Bethesda (MD): Eli Lilly & Company and the National Center for Advancing Translational Sciences; 2004-2013 May 1 [updated 2016 Jul 1].

[B42] Ryan M. J. Johnson G. Kirk J. Fuerstenberg S. M. Zager R. A. Torok-Storb B. (1994). HK-2: An immortalized proximal tubule epithelial cell line from normal adult human kidney. Kidney Int. 45 (1), 48–57. 10.1038/ki.1994.6 8127021

[B43] Sieber M. Hoffmann D. Adler M. Vaidya V. S. Clement M. Bonventre J. V. (2009). Comparative analysis of novel noninvasive renal biomarkers and metabonomic changes in a rat model of gentamicin nephrotoxicity. Toxicol. Sci. 109 (2), 336–349. 10.1093/toxsci/kfp070 19349640 PMC4830225

[B44] Sistare F. D. DeGeorge J. J. (2007). Preclinical predictors of clinical safety: Opportunities for improvement. Clin. Pharmacol. Ther. 82 (2), 210–214. 10.1038/sj.clpt.6100243 17507920

[B45] Sistare F. D. Dieterle F. Troth S. Holder D. J. Gerhold D. Andrews-Cleavenger D. (2010). Towards consensus practices to qualify safety biomarkers for use in early drug development. Nat. Biotechnol. 28 (5), 446–454. 10.1038/nbt.1634 20458314

[B46] Stepanenko A. A. Dmitrenko V. V. (2015). Pitfalls of the MTT assay: Direct and off-target effects of inhibitors can result in over/underestimation of cell viability. Gene. Elsevier B.V. 574 (2), 193–203. 10.1016/j.gene.2015.08.009 26260013

[B47] Tonomura Y. Tsuchiya N. Torii M. Uehara T. (2010). Evaluation of the usefulness of urinary biomarkers for nephrotoxicity in rats. Toxicology 273 (1–3), 53–59. 10.1016/j.tox.2010.04.015 20438795

[B48] Truong T. Suriyanarayanan T. Zeng G. Le T. Liu L. Li J. (2018). Use of Haploid Model of Candida albicans to Uncover Mechanism of Action of a Novel Antifungal Agent. Front. Cell. Infect. Microbiol. 8, 1–14. 10.3389/fcimb.2018.00164 29938200 PMC6002804

[B49] Vaidya V. S. Ozer J. S. Dieterle F. Collings F. B. Ramirez V. Troth S. (2010a). KIM1 iutperforms traditional biomarkers of kidney infury. Nat. Biotechnol. 28 (5), 478–485. 10.1038/nbt.1623.Kidney 20458318 PMC2885849

[B50] Vaidya V. S. Ozer J. S. Dieterle F. Collings F. B. Ramirez V. Troth S. (2010b). Kidney injury molecule-1 outperforms traditional biomarkers of kidney injury in preclinical biomarker qualification studies. Nat. Biotechnol. 28 (5), 478–485. 10.1038/nbt.1623 20458318 PMC2885849

[B51] Van Etten E. W. M. Van Den Heuvel-de Groot C. Bakker-woudenberg I. A. J. M. (1993). Efficacies of amphotericin b-desoxycholate (fungizone), liposomal amphotericin b (ambisome) and fluconazole in the treatment of systemic candidosis in immunocompetent and leucopenic mice. J. Antimicrobial Chemother. 32 (5), 723–739. 10.1093/jac/32.5.723 8125837

[B52] Varghese F. Bukhari A. B. Malhotra R. De A (2014). IHC profiler: An open source plugin for the quantitative evaluation and automated scoring of immunohistochemistry images of human tissue samples. PloS One 9 (5), e96801. 10.1371/journal.pone.0096801 24802416 PMC4011881

[B53] Varlam D. E. Siddiq M. M. Parton L. A. Rüssmann H (2001). Apoptosis Contributes to Amphotericin B- Induced Nephrotoxicity. Antimicrob. Agents Chemother. 45, 3, 679–685. 10.1128/AAC.45.3.679-685.2001 11181342 PMC90355

[B54] Vinken P. Starckx S. Barale-Thomas E. Looszova A. Sonee M. Goeminne N. (2012). Tissue Kim-1 and urinary clusterin as early indicators of cisplatin-induced acute kidney injury in rats. Toxicol. Pathol. 40 (7), 1049–1062. 10.1177/0192623312444765 22581811

[B55] Wadey R. M. Pinches M. G. Jones H. B. Riccardi D. Price S. A (2014). Tissue expression and correlation of a panel of urinary biomarkers following cisplatin-induced kidney injury. Toxicol. Pathol. 42 (3), 591–602. 10.1177/0192623313492044 23823703

[B56] Whaley S. G. Berkow E. L. Rybak J. M. Nishimoto A. T. Barker K. S. Rogers P. D. (2017). Azole antifungal resistance in Candida albicans and emerging non-albicans Candida Species. Front. Microbiol. 7 (JAN), 1–12. 10.3389/fmicb.2016.02173 PMC5226953 28127295

[B57] Wisplinghoff H. Bischoff T. Tallent S. M. Seifert H. Wenzel R. P. Edmond M. B. (2004). Nosocomial Bloodstream Infections in US Hospitals: Analysis of 24,179 Cases from a Prospective Nationwide Surveillance Study. Clin. Infect. Dis. 39 (3), 309–317. 10.1086/421946 15306996

[B58] Wong S. S. W. Kao R. Y. Yuen K. Y. Wang Y. Yang D. Samaranayake L. P (2014). In vitro and in vivo activity of a novel antifungal small molecule against Candida infections. PloS One 9 (1), e85836. 10.1371/journal.pone.0085836 24465737 PMC3899067

[B59] Yang L. Brooks C. R. Xiao S. Sabbisetti V. Yeung M. Y. Hsiao L. L (2015). KIM-1 – mediated phagocytosis reduces acute injury to the kidney Find the latest version : KIM-1 – mediated phagocytosis reduces acute injury to the kidney. J. Clin. Invest. 125 (4), 1620–1636. 10.1172/JCI75417 25751064 PMC4396492

[B60] Zhou Y. Vaidya V. S. Brown R. P. Zhang J. Rosenzweig B. A. Thompson K. L (2008). Comparison of kidney injury molecule-1 and other nephrotoxicity biomarkers in urine and kidney following acute exposure to gentamicin, mercury, and chromium. Toxicol. Sci. 101 (1), 159–170. 10.1093/toxsci/kfm260 17934191 PMC2744478

